# Mitochondria-Rich Cells: A Novel Type of Concealed Cell in the Small Intestine of Chinese Soft-Shelled Turtles (*Pelodiscus Sinensis*)

**DOI:** 10.3390/ani9100717

**Published:** 2019-09-24

**Authors:** Waseem Ali Vistro, Yifei Liu, Mengdi Xu, Ping Yang, Abdul Haseeb, Yufei Huang, Xuebing Bai, Liang Yu, Noor Samad Gandahi, Imran Tarique, Qiusheng Chen

**Affiliations:** MOE Joint International Research Laboratory of Animal Health and Food Safety, College of Veterinary Medicine, Nanjing Agricultural University, Nanjing 210095, Jiangsu, China; 2017207039@njau.edu.cn (W.A.V.); 2017807120@njau.edu.cn (Y.L.); 2017107004@njau.edu.cn (M.X.); yangping@njau.edu.cn (P.Y.); 2016207037@njau.edu.cn (A.H.); 2017207007@njau.edu.cn (Y.H.); 2016107003@njau.edu.cn (X.B.); 2016107004@njau.edu.cn (L.Y.); 2017207040@njau.edu.cn (N.S.G.); samoo_imran88@hotmail.com (I.T.)

**Keywords:** MRC, ultrastructure, Na^+^/K^+_^ATPase, Na^+^/K^+^/2Cl^−^ cotransporter, carbonic anhydrase, small intestine, Chinese soft-shelled turtle

## Abstract

Although some studies have been conducted over the past few decades, the existence of mitochondria-rich cells (MRCs) in reptiles is still obscure. This is the first study to uncover the presence of MRCs in the small intestine of Chinese soft-shelled turtles. In this study, we investigated the ultrastructural characteristics of MRCs and the secretion of different ion transport proteins in the small intestine of *Pelodiscus sinensis.* Transmission electron microscopy revealed that the ultrastructural features of MRCs are clearly different from those of other cells. The cytoplasmic density of MRCs was higher than absorptive epithelial cells (AECs) and goblet cells (GCs). MRCs possessed abundant heterogeneous mitochondria and an extensive tubular system in the cytoplasm, however, the AECs and GCs completely lacked a tubular system. Statistical analysis showed that the diameter and quantification of mitochondria were highly significant in MRCs. Mitochondrial vacuolization and despoiled mitochondria were closely associated with autophagosomes in MRCs. The multivesicular bodies (MVBs) and the exosome secretion pathway were observed in MRCs. Immunohistochemical staining of ion transport proteins indicated positive immunoreactivity of Na^+^/K^+_^ATPase (NKA) and Na^+^/K^+^/2Cl^−^ cotransporter (NKCC) at the basal region of the mucosal surface. Likewise, the immunofluorescence staining results showed a strong positive localization of NKA, NKCC, and carbonic anhydrase (CA) at the basal and apical region of the mucosal surface of small intestine. Our findings suggest that MRCs provide support and regulate cellular ions for intestinal homeostasis and provide energy for cellular quality control in intestine.

## 1. Introduction

The digestive system of reptiles consists of the same arrangement of digestive organs as the arrangement present in other higher vertebrates, from mouth to cloaca [1]. The reptilian small intestine is highly convoluted, and its surface is varied (longitudinal and transverse folds) among different groups, such as snakes and Nile monitors [2]. The small intestinal epithelial cells perform two essential functions, namely, the secretion and absorption of electrolytes (sodium, potassium, and chloride) and the movement of net fluid across the gastrointestinal epithelium, which is primarily the result of the active transport of different ions [3].

The lower intestinal epithelium is lined with simple columnar cells that contain cilia on their apical surface, and these cells are also known as dark cells or brush cells [4]. Some of these special ‘dark cells’ or ‘brush cells’ are classified as MRCs. MRCs are one of the most important epithelial cell types observed in the lower intestine of chickens and are located in the upper part of the mucosal folds. Localization of MRCs mainly has been reported in gills, kidney, intestine, and yolk-sac membrane of fish [5,6,7], as well as in chicken intestines [8]. The intestinal epithelium of the Chinese soft-shelled turtle is composed of AECs, GCs, intraepithelial lymphocytes (IELs), and plasma cells [9], however, to date, these MRCs have never been observed in the intestines of reptiles. 

Keys and Willmer were the first to explain the role of chloride cells in the secretion of Cl^−^ ions [10]. The function of these cells is not limited to the secretion of chlorine (Cl^−^); these cells also participate in the bidirectional transportation of many other ions, and it would be more suitable to refer to chloride cells as MRCs [11]. MRCs are a functional unit that regulate the ions in the teleost gill epithelium. These cells are morphologically characterized by numerous mitochondria with high electron density and contain numerous tubular systems in their cytoplasm [12]. Another morphological differentiation of MRCs is the development of multicellular complexes organized with adjacent accessory cells [11]. MRCs are the primary place for the active transport of ions in bronchial epithelial cells, and these cells secrete ions in seawater-adapted (SW-adapted) fish and in freshwater-adapted (FW-adapted) fish [13,14,15]. Certainly, amphibian skin is a heterogeneous epithelium, with MRCs scattered in the superficial layer among the bulk of the principal cells [16]. MRCs may also play an important role in acid–base regulation [17,18]. In addition, several environmental conditions, such as high temperature and high levels of organic matter, favor the occasional development of nitrite (NO_2_^2−^) in the Amazonian environment and, in fish culture systems, this energetic mechanism involves NO_2_^2−^ uptake by the MRCs in the gill [19]. MRCs in the kidney tubules and gills are the primary cells responsible for ion transport. These cells contain large quantities of Na^+^/K^+_^ATPase (NKA), which creates a driving force for ion uptake from the water by the lumen of renal tubules in the kidney and gills [20].

Several authors have reported that MRCs possess the cooperative action of different ion transport proteins, namely, NKA and Na^+^/K^+^/2Cl^−^ cotransporter (NKCC) [21]. Immunological localization studies have verified that NKA and NKCC are present at the basal region, and carbonic anhydrase (CA) is associated with the apical region with a tubular system of MRCs [22]. NKA is a widespread membrane-bound enzyme, which dynamically maintains the sodium potassium pump in animal cells. This protein is essential not only for the maintenance of intracellular homeostasis but also for impelling power for water and ion transport in the fish gills [23]. Fluctuation, like the high and low activity of the NKA pump, facilitates the absorption of nutrients within the small intestine [24]. NKA maintains low intracellular Na^+^ and high extracellular K^+^ concentrations, and NKCC mediates the entry of Na^+^ and Cl^−^ into the cellular compartment along the electrochemical gradient provided by NKA [25]. The Na^+^ gradient is used to transport Na^+^, K^+^ and 2Cl^−^ into the cell through a basolateral NKCC, and Cl^−^ then exits the cells along an electrical gradient through apical Cl^−^ channels [26]. CA is widely present in the intestine. Its physiological role is the absorption of salt, water, and acid–base homeostasis in the intestine [27], however, all of the above findings were observed in fish, avian, and amphibian species and there is no clear functional information related to MRCs in the small intestine of reptiles. Chinese soft-shelled turtle is one of the most nutritional and pharmacological worthy animals in China.

Therefore, the objective of this study is to investigate the possible morphological relationship and the ultrastructural characteristics of MRCs, as well as the secretion of different ion transport proteins by MRCs, in the small intestine of Chinese soft-shelled turtles.

## 2. Materials and Methods

### 2.1. Animals and Tissue Blocks Preparation

For the present study, ten mature (4–5 years) old, Chinese soft-shelled turtles (*Pelodiscus sinensis*) were purchased from an aquatic Nanjing farm, Jiangsu Province, China. The turtles were anaesthetized by intraperitoneal injection of sodium pentobarbital (20 mg/animal) and then killed by neck dislocation. The small intestine specimens were obtained quickly and fixed for different experimental processes (details blow). The process of sampling was approved by the college of Veterinary Medicine of Nanjing Agricultural University. The study procedure was approved by the Science and Technology Agency of Jiangsu Province (Approval ID: SYXK (SU) 2010-0005). All processes with turtles were performed according to the Animal Research Institute Ethics Committee guidelines of Nanjing Agricultural University, China.

### 2.2. Periodic Acid Schiff (PAS) Staining 

The fixed intestinal tissue slides were dehydrated with an alcohol grading sequence from 75–100%, with the samples incubated in each grade for 2 minutes and cleared in xylene for 10 minutes. Then, the tissue slides were incubated in periodic acid (5 g/L of water) for 5 minutes, rinsed in lukewarm water for 10 minutes, and stained in Coleman’s Schiff reagent for 10 minutes, and then in hematoxylin for a few seconds. The tissue slides were examined with an electron microscope (BX50; Olympus, Tokyo, Japan), and images of the tissue slides were taken with an air-conditioned control coupled apparatus camera (DP72; Olympus through Cell Sens software, Nanjing China).

### 2.3. Transmission Electron Microscopy (TEM)

After tissue sample collection, the tissues were cut into small pieces and immersed in 2.5% glutaraldehyde fixative in 0.1 M phosphate buffered saline (PBS) at 4 °C for 24 hours. The samples were post fixed in 1% (w/v) osmium tetroxide and washed in PBS three times. Dehydration was carried out with a graded series of ethanol (75–100%). Then, the samples were soaked in propylene oxide and embedded in Araldite. Ultrathin sections of selected areas were cut and mounted on Formvar-coated grids and stained with uranyl acetate and lead citrate for 20 minutes per step. The ultrastructure of the small intestine was viewed by a transmission electron microscope (Hitachi H-7650; Hitachi high-technologies Corporation, Tokyo, Japan). 

### 2.4. Immunohistochemistry (IHC)

A standard staining immunohistochemistry protocol for MRCs was performed according to previous studies [12]. Small intestinal tissue slides were deparaffinized in xylene two times each, for ten minutes. All slides were incubated in a graded series of ethanol (75–100%) for two minutes per grade. Antigenic sites were exposed by boiling for five minutes in 30% sodium citrate and then rinsed three times in phosphate buffered saline (PBS). Tissue expression was determined using a rabbit anti-NKA antibody (1:100) (Wuhan Xavier Biotechnology Co., Ltd, Wuhan, China). All sections were stored at 4 °C for one night. The next day, we used goat anti-rabbit IgG (SABC; Bio-sharp Biotechnology Co., Wuhan, China as the secondary antibody. Sections were colored with DAB (Diaminobenzidine) (Boster, Sigma Chemical Co., St. Louis, MO, China followed by counterstaining with hematoxylin. All the slides were incubated in distilled water followed by a graded series of ethanol. Finally, all the slides were processed by neutral balsam. For NKCC, the staining was performed as described above with a rabbit polyclonal antibody against NKA.

### 2.5. Immunofluorescence (IF)

After the removal of paraffin wax from tissue slides, tissue sections of the small intestine were incubated with primary antibodies against NKA, NKCC, and CA (1:100) at 4 °C overnight. Then, the slides were washed in PBS, an analogous secondary antibody was added, and sections were incubated for two hours at a normal temperature of 37 °C. Then, the sections were washed again with PBS, and 4’, 6’-diamino-2-phenylidole (DAPI) was used for nuclear staining. Images of the tissues were captured directly using an Olympus microscope (BX53) and camera (Olympus DP73, Olympus Corporation Company, Tokyo, Japan).

### 2.6. Statistical Analysis

The histomorphometry of stained tissue sections was measured by Image Pro v10 (International Scientific Community, Anaheim, CA, USA) and analyzed statistically with Graph-Pad Prism 7.0 software (IBMP Crop, USA). Available online: http://allpcworld.com/download-graphpad-prism-7-0-free/ (accessed on 17 May 2018). The results are presented as the mean ± SEM. The statistical significance of differences between the means was also analyzed by t-test (*p* ˂ 0.05).

## 3. Results

### 3.1. Light Microscopy

**Figure 1:**
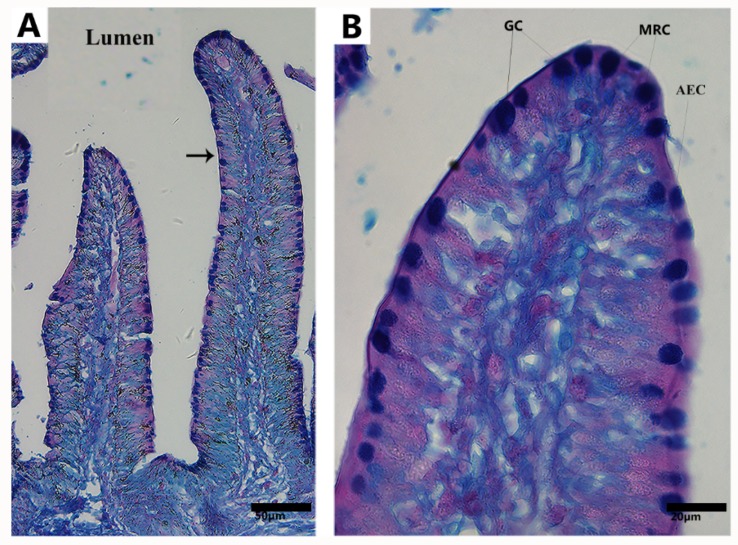
Light microscopy of the small intestine of *Pelodiscus sinensis*. Three main types of epithelial cells were present in the mucosal surface of the small intestine: mitochondria-rich cells (MRCs), absorptive epithelial cells (AECs), goblet cells (GCs), and villus (black arrow). Scale bars = (**A**) 50 µm and (**B**) 20 µm.

The small intestinal mucosa was composed of three main types of epithelial cells: (1) MRCs, (2) AECs, and (3) mucus secreting GCs (Figure 1). MRCs were very similar in shape and size to the AECs in the small intestine of Chinese soft-shelled turtles and were easily identified through light microscopy with high resolution.

### 3.2. Transmission Electron Microcopy

**Figure 2:**
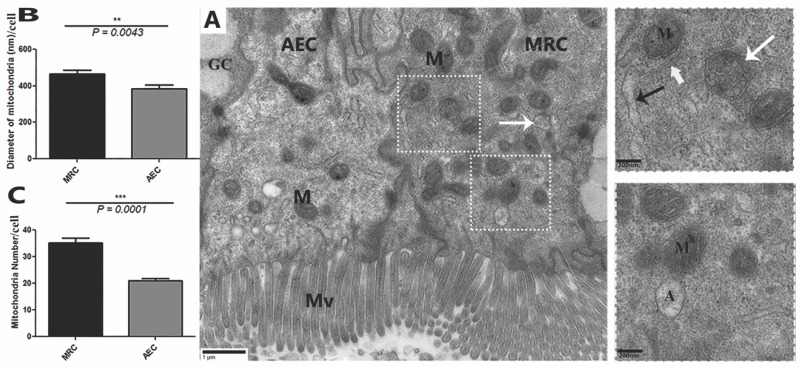
Transmission electron micrograph of the morphological comparison of mitochondria-rich cells (MRCs) with AECs and GCs of *P. sinensis*. (**A**) mitochondria-rich cell (MRC), absorptive epithelial cell (AEC), goblet cell (GC), mitochondria (M), autophagosome (A), microvillus (Mv), multivesicular bodies (white arrow), Golgi complex (thick white arrow) and endoplasmic reticulum (thin black arrow). (**B**) Quantification of the diameter of mitochondria and (**C**) number of mitochondria. Scale bar = (A) 1 µm.

**Figure 3:**
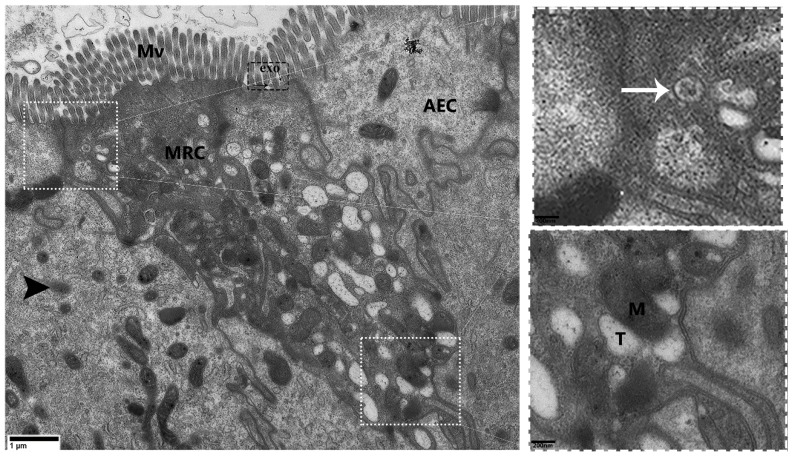
Transmission electron micrograph of an MRC that exhibits exosome secretion in the mucosal layer of *P. sinensis*. Mitochondria-rich cells (MRCs), absorptive epithelial cell (AEC), mitochondria (M), tubular system (T), multivesicular bodies (white arrow), exosome (exo), lysosome (black arrowhead), and microvilli (Mv). Scale bar =1 µm.

**Figure 4:**
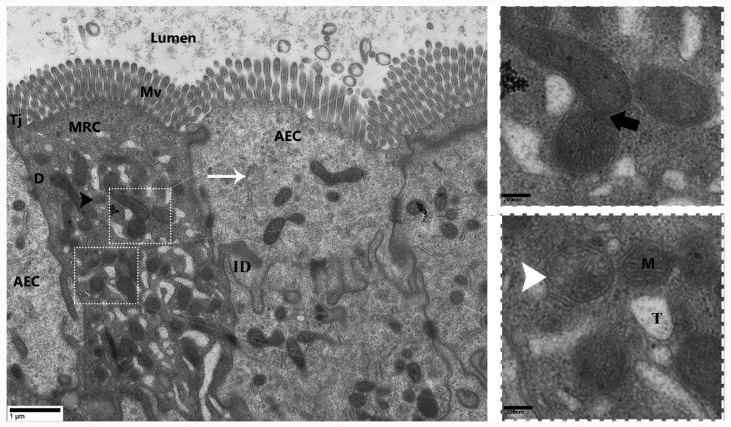
Transmission electron micrograph of MRC connected with the AEC in mucosal layer of *P. sinensis*. Mitochondria-rich cell (MRC), absorptive epithelial cell (AEC), tight junction (Tj), desmosome (D), interdigitation (ID), mitochondria (M), tubular system (T), multivesicular bodies (white arrow); vacuolization in mitochondria (white arrowhead), intermitochondrial junction (thick black arrow), lysosome (black arrowhead), microvilli (Mv). Scale bar = 1 µm.

Transmission electron micrograph provided strong evidence for the existence of MRCs in the small intestine of *P. sinensis.* These cells were especially located at the upper area of the lateral border of the mucosal folds. The cytoplasm of MRCs exhibited more electron density than that of AECs and GCs (Figure 2A). Most of the cytoplasm of the MRCs was occupied by mitochondria and an extensive tubular system. However, the cytoplasm of the AECs and GCs displayed a limited number and size of mitochondria. Notably, the cytoplasm of AECs and GCs lacked an extensive tubular system. However, statistical analysis of the diameter and quantification of the mitochondria in the MRCs showed that these features were highly significant as compared with those in the AECs (Figure 2B,C). Additionally, heterogeneous morphological mitochondria, endoplasmic reticulum, and dilated Golgi complexes were found in the MRCs, as well autophagic vacuoles similar to the mitophagic profile were recorded (Figure 2A). Moreover, the extensive tubular system of MRCs was directly interposed in the mitochondria and was continuous with the basolateral membrane, resulting in a large surface area for the placement of ion-transport proteins. In highly activated MRCs, the mitochondria were well developed, and some of them had low and high electron densities (Figure 3). Additionally, inside the cytoplasm of MRCs, membrane-bound, round structures knows as the multivesicular bodies (MVBs) appeared with low and high electron density. These MVBs were located near the plasma membrane and secreted exosomes which were present in the lumen of the small intestine (Figure 2A and Figure 3). The MRCs contain microvilli of various sizes, and extensive uniform interdigitation for linking with neighbor cells (AECs and GCs) was present around the lateral borders of the mucosal surface, increasing in complexity towards the base of the cells. The MRCs also played another distinct role in the maintenance of the physical barrier, as they were directly connected with AECs through tight junctions and desmosomes. In the MRCs, which showed morphological variation in mitochondria, we inferred that the increase in size was due to the fusion of mitochondria. Mitochondrial vacuolization and intermitochondrial junctions (IMJs) were observed within the MRCs (Figure 4). Furthermore, we have summarized the cytological features of MRCs, AECs, and GCs in the small intestine of Chinese soft-shelled turtles (Table 1). 

### 3.3. Immunohistochemistry and Immunofluorescence

**Figure 5:**
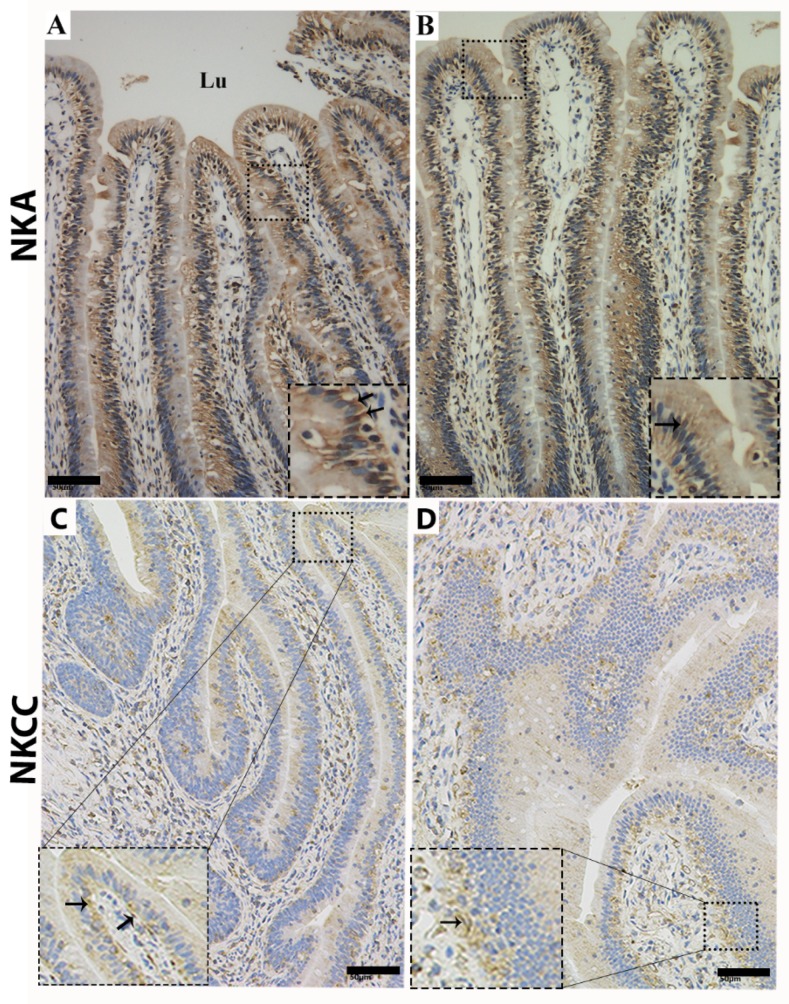
Immunohistochemical localization of Na^+^/K^+_^ATPase (NKA) and Na^+^/K^+^/2Cl^−^ cotransporter (NKCC) in the mucosal surface of the small intestine of *P. sinensis*. NKA and NKCC immunoreactivity showed positive expression in the mucosal surface (black arrow). Lu: lumen. Scale bar = (**A**–**D**) 50 µm.

**Figure 6:**
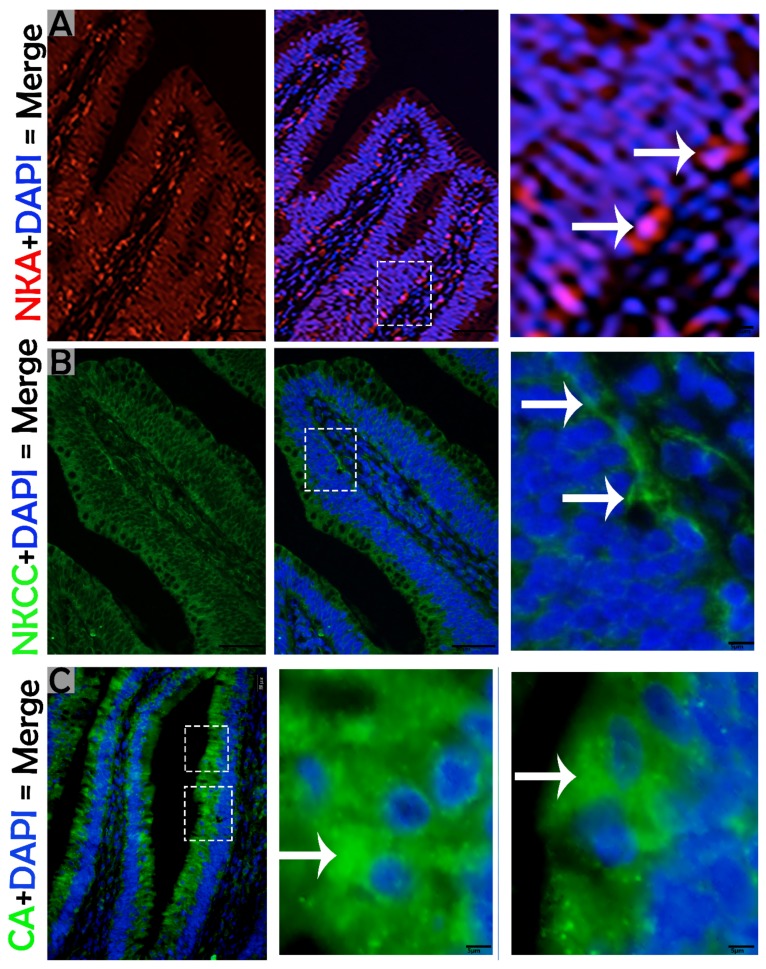
Immunofluorescence labeling of NKA, NKCC, and carbonic anhydrase (CA) in the small intestine of *P. sinensis*. The immunopositive localization of NKA, NKCC, and CA in the mucosal surface (white arrow). Scale bar = (**A**–**C**) 20 µm.

To investigate the ion transport proteins of MRCs in the mucosal surface of the small intestine, we used immunohistochemical staining to determine the expression of NKA and NKCC in MRCs. NKA and NKCC had more positive immunoreactivity at the basolateral zone of the mucosal surface of the small intestine (Figure 5A,C,D), and weak immunoreactivity was detected at the apical region of the mucosal surface (Figure 5B). Likewise, IF staining was performed to determine the cellular localization of NKA, NKCC, and CA. Immunofluorescence staining showed the strong positive immunostaining of NKA and NKCC at the basolateral region of the mucosal surface (Figure 6A,B), however, CA showed strong positive immunostaining at the apical region of the mucosal surface of the small intestine (Figure 6C). Overall, analysis of ion transport proteins by IHC and IF indicated that in the MRCs of the small intestine of *P. sinensis* the intensity of immunoreactivity increased at the basal and apical regions of the mucosal surface. 

## 4. Discussion

This study revealed, for the first time, the existence of MRCs within the small intestine of reptiles. Previously, these cells were revealed in the lower intestinal epithelium of chicken [8], the epithelial cells of the gills and kidney of fish [12,20,28,29], and the epidermis of the skin of amphibian [30]. Earlier studies showed that MRCs exist in clusters that form within the upper part of the mucosal fold. The cytoplasmic organelles of MRCs are very typical for ion transportation, while mitochondria and an extensive tubular system were more frequent than those in AECs [4]. In our results, MRCs were characterized by the occurrence of an abundant quantity of mitochondria with high electron density in the cytoplasm relative to AECs and GCs. The intestinal epithelium of the chicken contains an abundance of mitochondria, i.e., 19% in MRCs and 10% in AEC [31]. In contrast, in our study, mitochondria were present at 35% in MRCs and at 20% in AECs. In the present study, MRCs secreted different kinds of MVBs and exosomes with low and high electron density. A previous study observed that the luminal exosomes released from murine IEC4.1 help with epithelial antimicrobial protection [22], and therefore we assumed that the MRCs of turtle may perform similar immune functions.

Furthermore, these cells are differentiated by an extensive tubular system in the cytoplasm, which is distributed throughout the cytoplasm and directly interposed within the mitochondria for the placement of ion-transport proteins (NKA and NKCC). In fresh water milkfish, the tubular system of MRCs is dense along the basolateral membrane but more aggregated and very small in size around the mitochondria [28]. As shown by Luca and Towel [32], in MRCs, the tubular system increases its dispersion throughout the cytoplasm, except for the subapical region, to enlarge the surface area through which Na^+^ and K^+^ are transported. NKA is a main enzyme in the ion transportation of MRCs, which creates ionic and electronic gradients for ion secretion and absorption [33]. Immunocytochemistry labeling and electron microscopic observation of tilapia showed that NKA and NKCC are present in the basolateral membrane of the tubular system of MRCs [34] and similar immunofluorescence staining results for NKA and NKCC were observed in the present study. MRCs are the sites for active ammonia elimination through the ion transport proteins, NKA and NKCC [35]. In the intestinal epithelium, the activity of NKA pumps helps to achieve a substantial portion of the total energy consumption by tissue [31]. Additionally, we observed two types of mitochondria in MRCs which were distinguished by their electron density and were adjoined to form intermitochondrial junctions (IJMs). MRCs were directly connected with GCs and AECs through laterally extensive interdigitation and, consequently, developed physical barriers (desmosomes and tight junctions) for the support and maintenance of the internal milieu of the mucosal surface. MRCs are highly reactive and these cells contain carbonic anhydrase, which plays a significant role in acid–base transport for homeostasis in mammalian collecting duct MRCs [36]. The presence of CA in the branchial MRCs of fish gills indicates their involvement in the elimination of ammonia and in the active elimination of Cl^−^ [37]. MRCs proliferate to increase the ion regulatory capacity of the fish gills in response to osmotic challenges. *Colisalalia* and *Trichigastertrichopterus* are both primary freshwater fishes with labyrinths in their gill chambers to enhance air breathing [29]. Chinese soft-shelled turtles hibernate in water for a long time and, consequently, require more ATP utilization for their continued existence. In addition, we observed autophagic vacuoles in MRCs and electron dense contents, similar to autophagy. Intestinal epithelial cell autophagy protects against tissue invasion by both opportunistically hostile commensals and invasive intestinal pathogens [38]. In a previous report, autophagy maintained cellular function and was involved in the elimination of damaged mitochondria [39]. In the current study, cristealysis and matrix vacuolization of mitochondria in MRCs were observed, and the mitochondria appeared as fluid-filled spherical vacuoles. Meanwhile, the endoplasmic reticulum and dilated Golgi complex were spread around the mitochondria. Many studies have demonstrated the contribution of the endoplasmic reticulum (ER) to autophagy vacuole formation and the interchange of molecules with mitochondria for the maintenance of cellular homeostasis. Mitophagy may be associated with mitochondrial fission by separating functional mitochondria from the damaged portion [39]. Additionally, after nitrite treatment, the numbers of mitochondria-rich cells decreased, and swelling of the endoplasmic reticulum and vacuolization of the mitochondria occurred inside the mitochondria-rich cells [19].

## 5. Conclusions

**Figure 7:**
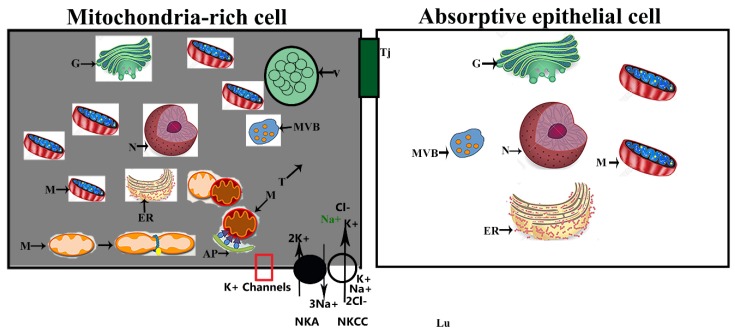
Schematic diagram of morphology and functional activity of MRC in the small intestine of *P. sinensis*. Dense cytoplasm MRCs contain greater numbers of mitochondria and a tubular system compared to absorptive epithelial cells. Cl^−^ enters MRCs via a Na^+^/K^+^/2Cl^–^ cotransporter (NKCC) driven by the Na^+^ gradient, which is maintained by Na^+^/K^+^_−_ATPase (NKA). The red rectangle highlights the potassium channels required in this process. Nucleus (N), mitochondria (M), endoplasmic reticulum (ER), autophagosome (AP), tubular system (T), multivesicular bodies (MVB), vacuolization (V), tight junction (Tj), and Golgi complex (G).

In this study, we provided new insight regarding the existence of MRCs in the small intestine of Chinese soft-shelled turtles. These cells are very distinct in morphology and ultrastructure, and their electron density clearly separates them from the AECs and GCs. A schematic diagram of the morphology and functional activity of MRCs in the small intestine of *P. sinensis* is presented in (Figure 7). This study highlights the significance of the ultrastructure and the different ion transport proteins of MRCs in the small intestine. However, the immunological role of MRCs in the intestine needs more exploration.

## Figures and Tables

**Table 1:** Summary of the distribution of cytological features of MRC, AEC, and GC in the mucosal layer of small intestine of Chinese soft-shelled turtle (n = 3).

Cytological Parameters	MRC	AEC	GC
Cytoplasmic density	+++	+	+
**Mitochondria**			
Overall amount	+++	+	+
Morphological heterogeneity	++	++	++
Vacuolization	+	−	−
MVB	+	+	−
Lysosome	+	+	+
**Tubular system (TS)**			
Dilated TS	+++	−	−
Small TS	++	−	−

Coding: −, absent; +, little; ++, moderately; +++, strongly.
